# Effect of the bacterial community assembly process on the microbial remediation of petroleum hydrocarbon-contaminated soil

**DOI:** 10.3389/fmicb.2023.1196610

**Published:** 2023-05-25

**Authors:** Xuehao Zheng, Belay Tafa Oba, Chenbo Shen, Luge Rong, Bin Zhang, Ling Huang, Lujie Feng, Jiani Liu, Tiantian Du, Yujie Deng

**Affiliations:** ^1^School of Geographical Sciences, China West Normal University, Nanchong, China; ^2^Sichuan Provincial Engineering Laboratory of Monitoring and Control for Soil Erosion in Dry Valleys, China West Normal University, Nanchong, China; ^3^Liangshan Soil Erosion and Ecological Restoration in Dry Valleys Observation and Research Station, Xide, China; ^4^College of Natural Science, Arba Minch University, Arba Minch, Ethiopia; ^5^Department of Civil and Environmental Engineering, Carnegie Mellon University, Pittsburgh, PA, United States; ^6^School of Biomedical and Chemical Engineering, Liaoning Institute of Science and Technology, Benxi, China

**Keywords:** petroleum hydrocarbons-contaminated soil, bacterial community assembly process, soil remediation, microbial remediation, surfactant

## Abstract

**Introduction:**

The accumulation of petroleum hydrocarbons (PHs) in the soil can reduce soil porosity, hinder plant growth, and have a serious negative impact on soil ecology. Previously, we developed PH-degrading bacteria and discovered that the interaction between microorganisms may be more important in the degradation of PHs than the ability of exogenous-degrading bacteria. Nevertheless, the role of microbial ecological processes in the remediation process is frequently overlooked.

**Methods:**

This study established six different surfactant-enhanced microbial remediation treatments on PH-contaminated soil using a pot experiment. After 30 days, the PHs removal rate was calculated; the bacterial community assembly process was also determined using the R language program, and the assembly process and the PHs removal rate were correlated.

**Results and discussion:**

The rhamnolipid-enhanced *Bacillus methylotrophicus* remediation achieved the highest PHs removal rate, and the bacterial community assembly process was impacted by deterministic factors, whereas the bacterial community assembly process in other treatments with low removal rates was affected by stochastic factors. When compared to the stochastic assembly process and the PHs removal rate, the deterministic assembly process and the PHs removal rate were found to have a significant positive correlation, indicating that the deterministic assembly process of bacterial communities may mediate the efficient removal of PHs. Therefore, this study recommends that when using microorganisms to remediate contaminated soil, care should be taken to avoid strong soil disturbance because directional regulation of bacterial ecological functions can also contribute to efficient removal of pollutants.

## 1. Introduction

Leakage incidents from petroleum production and transportation are inevitable. Petroleum hydrocarbons (PHs) entering the soil can reduce soil porosity and hinder plant growth (Feng et al., 2021). Benzenes and polycyclic aromatic hydrocarbons are typical PHs having strong teratogenic, carcinogenic, and mutagenic effects on soil ecology and human health (Wu et al., 2016; Zheng et al., 2020; Rong et al., 2021).

Exogenous microorganisms can be used to remediate petroleum hydrocarbon-contaminated soil at low cost and with high operability, but their impact on engineering applications is minimal (Feng et al., 2021; Rong et al., 2021; Liu et al., 2022a). Some intensification methods, such as looking for plants to provide root attachment sites for exogenous microorganisms (Zheng et al., 2020), adding nutrients to enable the rapid proliferation of exogenous microorganisms in the soil (Wang et al., 2017), and adding surfactants to increase the bioavailability and microbial activity, can effectively improve the remediation of PHs using microorganisms (Sun et al., 2018; Wang et al., 2018).

Researchers have discovered that the assembly process of microbial communities is influenced by deterministic or stochastic processes as microbial ecology has evolved (Ning et al., 2020). While there is no doubt that microbial assembly processes have a significant impact on organic pollutant bioremediation, studies on the correlation between microbial community assembly and organic degradation are rare. Recent studies have shown that the degradation of TPHs depends more on the interactions among microorganisms than it does on the potential of exogenous-degrading bacteria (Rong et al., 2021), which enriches the traditional understanding of bioremediation of contaminated soil, and also implies that microbial community's assembly may affect pollutant removal. From the points raised above, what are the differences among various treatments in the assembly processes of microbial community structures? and can the process of assembling a microbial community structure result in PHs degradation? In this study, the bacterial assembly process based on bacterial community structure data was calculated, and the correlation between the bacterial assembly and PHs degradation rate was analyzed.

## 2. Methods and materials

### 2.1. Sampling and the remediation process

The contaminated soil was collected from Xinmin petroleum field, Xinmin, Shenyang City, Liaoning Province (123°5′27″, 41°46′33″). The topsoil (0–20 cm) was collected and stored in a dark place for backup after being cleared of debris. The original TPH pollution level was 2,750 mg/kg.

The remediation process which has been described in Rong et al. (2021) is briefly summarized as follows: *Bacillus methylotrophicus* (bacteria N) and *Bacillus subtilis* (bacteria Y) have been carefully chosen as effective soil PH degradation bacteria. According to studies on the toxicity of surfactants to bacteria (Wang et al., 2018; Zheng et al., 2020), a total of six treatments were conducted on 10 kg soil with different exogenous bacteria and surfactants, including (1) CK: no reagent added; (2) N+RL: containing 1 L of N bacterial suspension (10^8^ CFU per milliliter of bacteria) and a rhamnolipid (RL) solution to achieve a rhamnolipid concentration of 500 mg/kg in the soil; (3) Y+RL: comprising 1 L of Y bacteria suspension and rhamnolipid solution to make the rhamnolipid concentration in the soil reach 500 mg/kg; (4) N+Y+RL: containing 1 L of N bacterial suspension and 1 L of Y bacterial suspension, and rhamnolipid solution to make the concentration of rhamnolipid in the soil reach 500 mg/kg; (5) N+Tween 80: comprised of 1 L of N bacterial suspension and polyethylene glycol sorbitan monooleate (Tween 80) solution to make the rhamnolipid concentration in the soil reach 2,000 mg/kg; (6) Y+SDBS: made of 1 L of Y bacterial suspension and sodium dodecyl benzene sulfonate (SDBS) solution to make the rhamnolipid concentration in the soil reach 3,000 mg/kg. Each treatment was carried out in triplicate.

**Figure 1:**
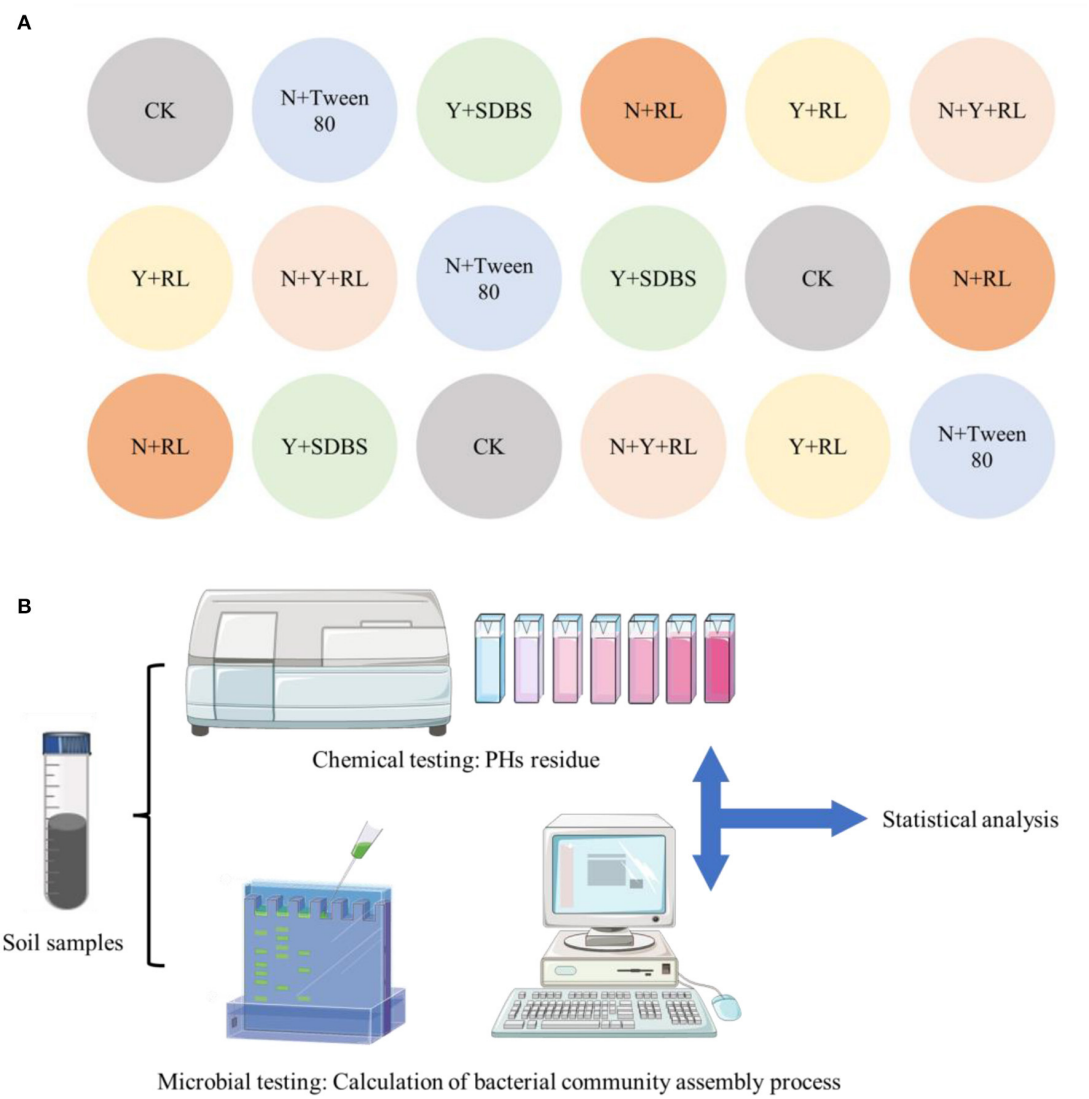
**(A)** Arrangement of the pots. A total of six treatments, each with three replicates, all pots randomly placed outdoors; **(B)** experimental process.

The pot-based remediation experiment was carried out at Shenyang University. To prevent cross contamination, all flower pots were randomly placed (Figure 1A), with a 20 cm space between each pot. Outdoor weather conditions were reported by Rong et al. (2021). After 30 days, the soil samples in the pot were mixed under sterile conditions, as shown in Figure 1B, for chemical and microbiological testing. The PHs in the soil were measured using an infrared oil meter (Wu et al., 2016). The bacterial community structure in the remediated soil was analyzed using 16S rRNA technology, and the testing process was conducted at Meiji Biotechnology Co., Ltd. The testing process referred to the standard methods issued by the testing company. The sequencing results were uploaded to the SRA database (SUB8645279). After calculating the assembly process of bacterial communities, Pearson's correlation analysis was conducted between the assembly process parameters and the removal rate of PHs.

### 2.2. Calculation of nearest taxon index

MEGA (Version 5.05) was used to build the phylogenetic trees. Using the “ape” and “picante” packages in R (Version 4.1.3) to quantify the mean nearest taxon distance (MNTD) and nearest taxon index (NTI) of microbial communities within a single sample, MNTD calculates the phylogenetic distance between species within a community. MNTD can find the phylogenetic distance between each OTU in the sample and the phylogenetic distance between its closest relatives and then obtain a weighted average of abundance on these phylogenetic distances. NTI is a standardized measure of the phylogenetic distance from each taxon in the sample to the nearest taxon, quantifying the degree of terminal clustering (Zhao et al., 2021). The calculation and analysis procedures were as follows:

(i) Input phylogenetic tree and species abundance table.

(ii) Find the phylogenetic distance between each species in the sample and the phylogenetic distance between their closest relatives. Calculate the weighted average of the phylogenetic distance and relative abundance according to Equation (1) to output MNTD_obs:_


(1) $$\begin{array}{l} MNTD_{obs}=\sum_{i_{k}=1}^{n_{k}}f_{i_{k}}min\left({△}_{i_{k}j_{k}}\right)\;\left(1\right) \end{array}$$

where *f*_*i*_*k*__ is the relative abundance of species i in the community k, n_k_ is the number of species in the community k, and *min*(_*i*_*k*_*j*_*k*__) is the minimum phylogenetic distance between species i and other species j in the community k (Anderson et al., 2011; Stegen et al., 2012).

(iii) After stochastic assigning each species and their relative abundance at each tip of phylogeny for 1,000 times, the MNTD value of the stochastic community is obtained as MNTD_null_. The mean MNTD (meanMNTD_null_) and standard deviation MNTD_null_ (sdMNTD_null_) are calculated, and the NTI was calculated according to the formula Equation (2):


(2) $$\begin{array}{l} NTI=\frac{mean\left(MNTD_{null}\right)-MNTD_{obs}}{sd\left(MNTD_{null}\right)}\;\left(2\right) \end{array}$$

(iv) NTI > 0 indicates that the relationship between coexisting species is closer than expected, and the system undergoes physiological clustering, with a deterministic process dominating structural changes within the community; NTI < 0 indicates that the relationship between coexisting species is farther than expected, and the system is phylogenetic stochastic, with stochastic processes leading to structural changes within the community (Feng et al., 2018). The difference between NTI and 0 represents the degree of clustering or dispersion of the system, that is, the impact of deterministic or stochastic processes on structural changes within a community.

### 2.3. Calculation of beta nearest taxon index

Beta mean nearest taxon distance (βMNTD) and beta nearest taxon index (β-NTI) were used to reflect changes in system development over time or space and were seen as an inter-group analog of MNTD and NTI (Fine and Kembel, 2011).

The calculation formula of βMNTD_obs_ was shown in Equation (3):


(3) $$\beta MNTD_{obs}=0.5\left[\sum_{i_{k}=1}^{n_{k}}f_{i_{k}}min{(}_{i_{k}j_{m}})+\sum_{i_{m}=1}^{n_{m}}f_{i_{m}}min(\Delta_{i_{m}j_{k}})\right]\;$$

where Δ_*i*_*k*_*j*_*m*__ is the minimum phylogenetic distance between species i in community k and species j in community m. The other variables are the same as those in Equation (1).

When β-NTI > 2 or β-NTI < −2, it indicates that the actual phylogenetic turnover between two communities is higher or lower than the expected phylogenetic turnover, i.e., the deterministic process dominates the structural changes; when −2 < β-NTI < 2, it indicates that the actual phylogenetic turnover between the two communities is similar to the expected phylogenetic turnover, i.e., the stochastic process dominates the structural changes (Anderson et al., 2011; Zheng et al., 2022). The difference between |β-NTI| and 0 represents the degree of clustering or dispersion of the system, that is, the impact of deterministic or stochastic processes on structural changes within a community.

### 2.4. Null model analysis

To further quantify the effect of deterministic or stochastic processes on changes in the structure of communities, a null model calculation and analysis were performed using the difference and similarity index between communities calculated based on the Bray–Curtis distance (Liu et al., 2022b). The specific steps followed were as follows:

(i) The Bray–Curtis distance calculated using the 'vegan' package was used as an index to characterize the similarity difference between communities, expressed in D_obs_. The range of D_obs_ is 0–1, and the closer the D_obs_ to 1, the greater the difference between the two communities; the similarity index between communities was expressed as S_obs_, in which S_obs_ = 1–D_obs_, the closer the S_obs_ to 1, the greater the similarity between the two communities. The calculated average value was $\bar{S_{obs}}$.

(ii) Using the randomize Matrix function in the “picante” package of R, keep the frequency of each species constant, randomly allocate the species abundance in each community, calculate the similarity index S_null_ between randomly distributed communities in the null model, and repeat the process 1,000 times to obtain the average value of the similarity index $\bar{S_{null}}$.

(iii) Permutational multivariate analysis of variance is a multivariate analysis of variance based on distance matrices. It uses Perm ANOVA to perform a significant difference analysis of the similarity matrix between the actual microbial community and the randomly distributed microbial community with a null model. If *P*-value is < 0.05, it indicates that there is a significant difference between the actual community and the randomly distributed community in the null model, that is, the deterministic process dominates the community. On the contrary, if the stochastic process dominates the community, there is no significant difference between the actual community and the null model randomly distributed community.

(iv) According to the difference between the similarity index obtained from the null model and the actually observed community, which accounts for the proportion of the actually observed community similarity index, the proportion of the impact of the deterministic process in community construction can be quantified and expressed as the deterministic ratio (DR), $DR=\left(\bar{S_{obs}}-\bar{S_{null}}\right)/\bar{S_{obs}}$; the impact ratio of stochastic processes in community construction is expressed as the stochastic ratio (SR), *SR* = 1−*DR* (Chase et al., 2011; Zhang et al., 2019).

### 2.5. Statistical analysis

Experimental data were analyzed using Excel (Version 2016). GraphPad Prism (Version 8.3.0) was used in the data graphing. Experimental data were presented as the mean and standard error. The significance of the differences was determined using Student's *t*-test and one-way ANOVA. The statistical analyses were performed using SPSS (Version 27).

## 3. Results

### 3.1. PHs removal rate

**Figure 2:**
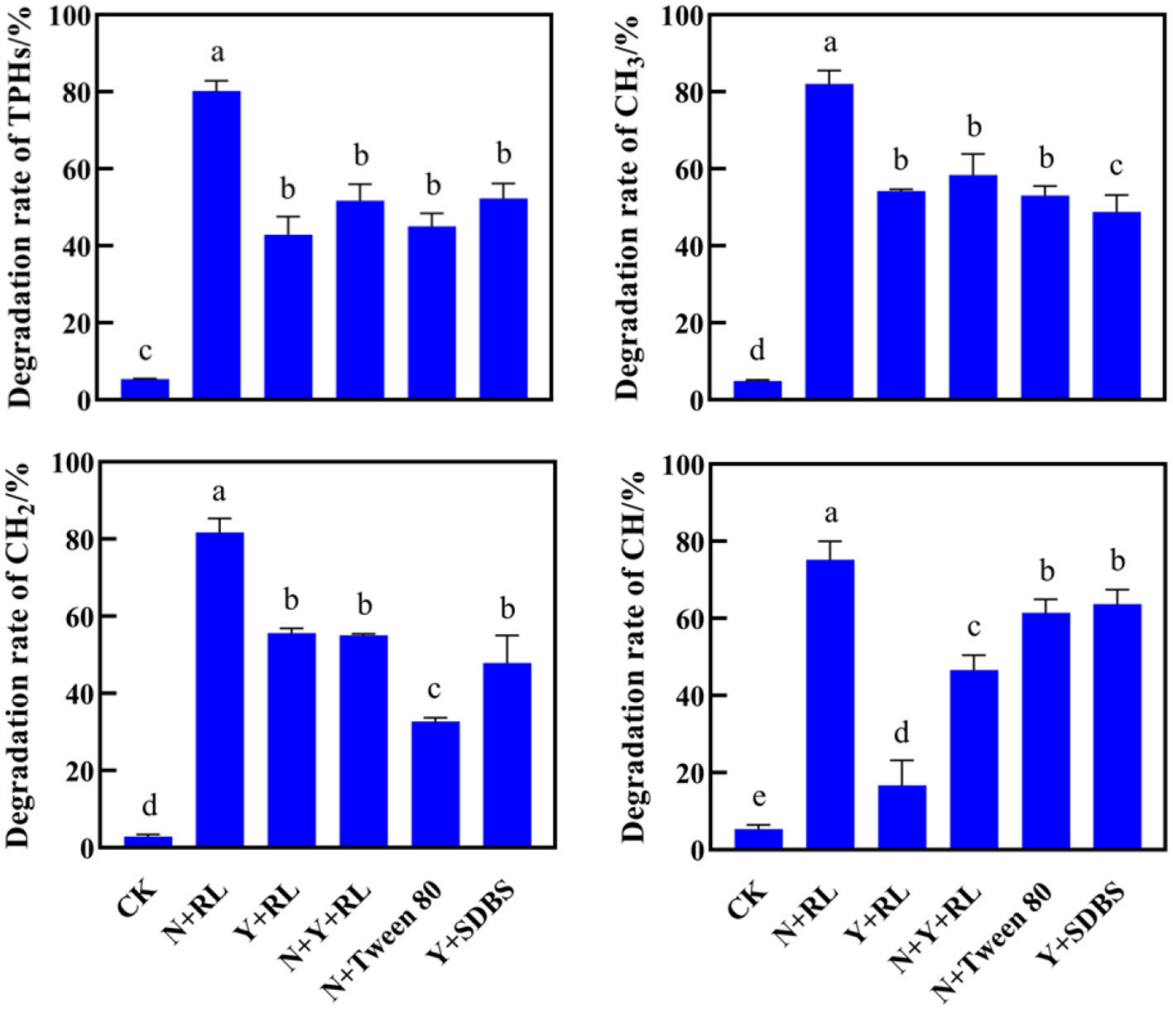
Degradation rates of total petroleum hydrocarbons (TPHs), alkanes (CH_3_), olefins (CH_2_), and aromatics (CH) by different treatments. The letters on the error bar (mean ± sd) indicate the results of the difference analysis (one-way ANOVA).

After 30 days, the removal rates of PHs by different treatments are shown in Figure 2. Data on the residual amount of PHs were published by Rong et al. (2021). The natural degradation rate of PHs in the soil was very low, only below 5%. The effect of N+RL treatment was found to be the best, with a removal rate of 80.24% for TPHs, and 82.03, 81.75, and 75.18% for alkanes (CH_3_), olefins (CH_2_), and aromatics (CH), respectively. There was no statistically significant difference in the TPH removal rates among Y + RL, N + Y + RL, N + Tween 80, and Y + SDBS (*P* > 0.05). The effects of various treatments on CH removal vary greatly. The ring-opening process involved in CH degradation required a large amount of energy. It was difficult to determine the main influencing factors related to pollutant removal from the analysis of bacterial species and surfactant species.

### 3.2. Assemble process analysis

This study chose the species with relative abundance >0.2% (RA > 0.2%) and RA > 0.5% in each sample for calculation and analysis because a total of 6,028 species were checked out in the sequencing results, the sequence file was too large to run when creating an evolutionary tree using MEGA (Version 5.05), and the majority of the species were rare species with relative abundance < 0.01%. The two microbial groups account for 85 and 75% of total abundance, respectively, and may well-represent the core species that play a significant role in community formation.

**Figure 3:**
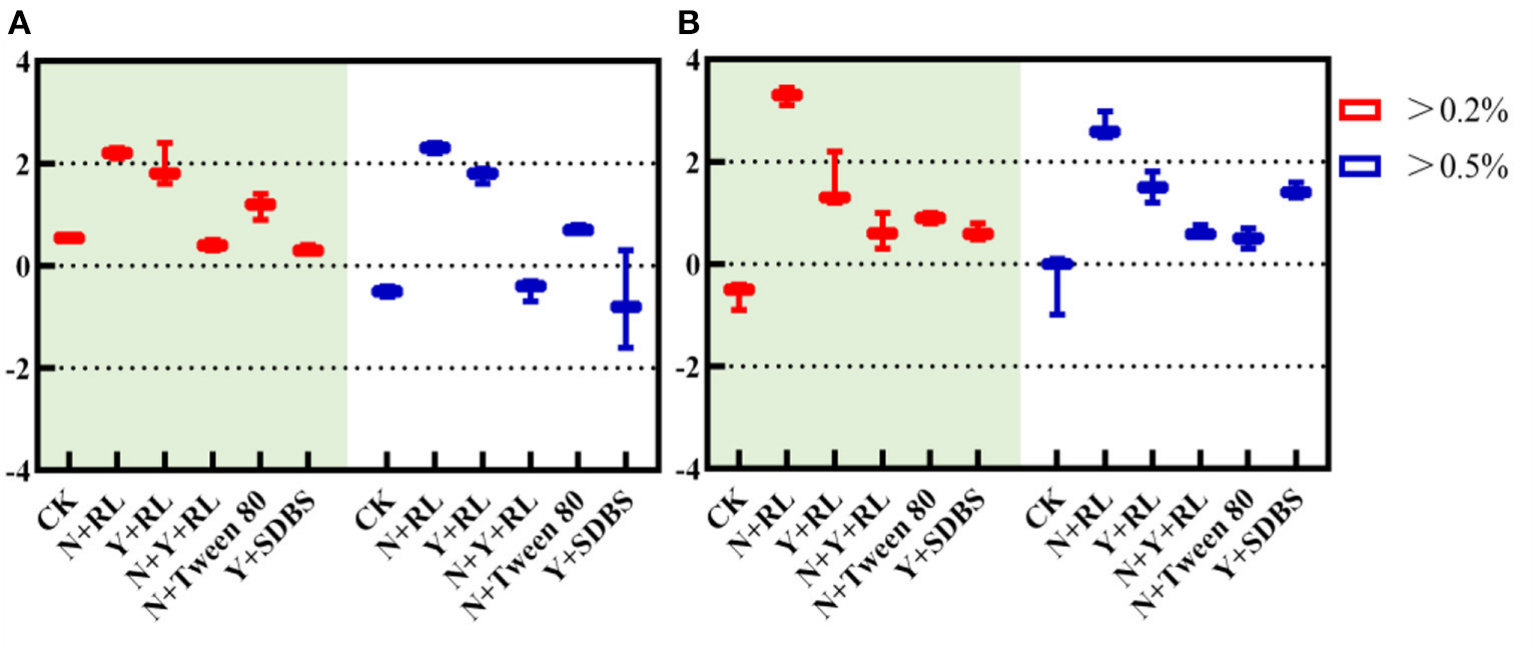
**(A)** Nearest taxon index (NTI) and **(B)** β nearest taxon index (β-NTI) in different abundance ranges.

As can be seen in Figure 3A, species with RA > 0.2% in all treatments have an $\bar{NTI}\;>\;0$, indicating that clustering occurred in all systems, and deterministic processes dominated community assembly; when RA > 0.5%, the $\bar{NTI}\;>\;0$ in N + RL, Y + RL, and N + Tween 80 indicates that clustering occurs in the system, and deterministic processes dominate community formation.

As can be seen in Figure 3B, when RA > 0.2%, the N + RL with β-NTI > 2 indicates that the phylogenetic turnover between communities was greater than the expected phylogenetic turnover. The deterministic process dominates the structural changes when the CK, Y + RL, N + Y + RL, N + Tween, and Y + SDBS have a mean β-NTI between −2 and 2, indicating that the phylogenetic turnover between communities was similar to the expected one, and the stochastic process dominates the structural changes.

Combined with NTI and β-NTI, for core communities with high relative abundance, the community of N + RL is assembled with a deterministic process, while the CK, Y + RL, N + Y + RL, N + Tween, and Y + SDBS are assembled with a stochastic process.

### 3.3. Null model fitting

The null model fitting results are shown in Table 1. There was no significant difference between the $\bar{S_{obs}}$ and $\bar{S_{null}}$ in CK, N + Y + RL, and N + Tween 80 (*P* > 0.05), indicating that the impact of the stochastic process on the community structure assembly of the three treatments was greater than that of deterministic processes. There was a significant difference between $\bar{S_{obs}}$ and $\bar{S_{null}}$ in the N + RL, Y + RL, and Y + SDBS, indicating that the deterministic assembly process was dominant (*P* < 0.05).

**Table 1:** Null model calculation results based on Bray–Curtis distance.

**Treatment**	** $\bar{S_{obs}}$ **	** $\bar{S_{null}}$ **	***P*-value**	**DR**	**SR**
CK	0.4201	0.3438	0.2428	18.17%	81.83%
N + RL	0.7363	0.2459	0.0010	66.60%	33.40%
Y + RL	0.4533	0.2352	0.0439	54.86%	45.14%
N + Y + RL	0.5848	0.3657	0.0629	37.46%	62.54%
N + Tween 80	0.5019	0.3660	0.1518	27.09%	72.91%
Y + SDBS	0.5303	0.2562	0.0160	51.69%	48.31%

After quantifying the impact of deterministic processes on community assembly processes using DR, the results showed that the DR of CK, N + Tween, and N+Y+RL was < 50%, indicating that stochastic assembly processes lead the structural change. The DR of N + RL, Y + RL, and Y + SDBS was >50%, indicating that the structural change was caused by the deterministic assembly process.

### 3.4. Effect of bacterial community assembly process on the rate of PHs removal

**Figure 4:**
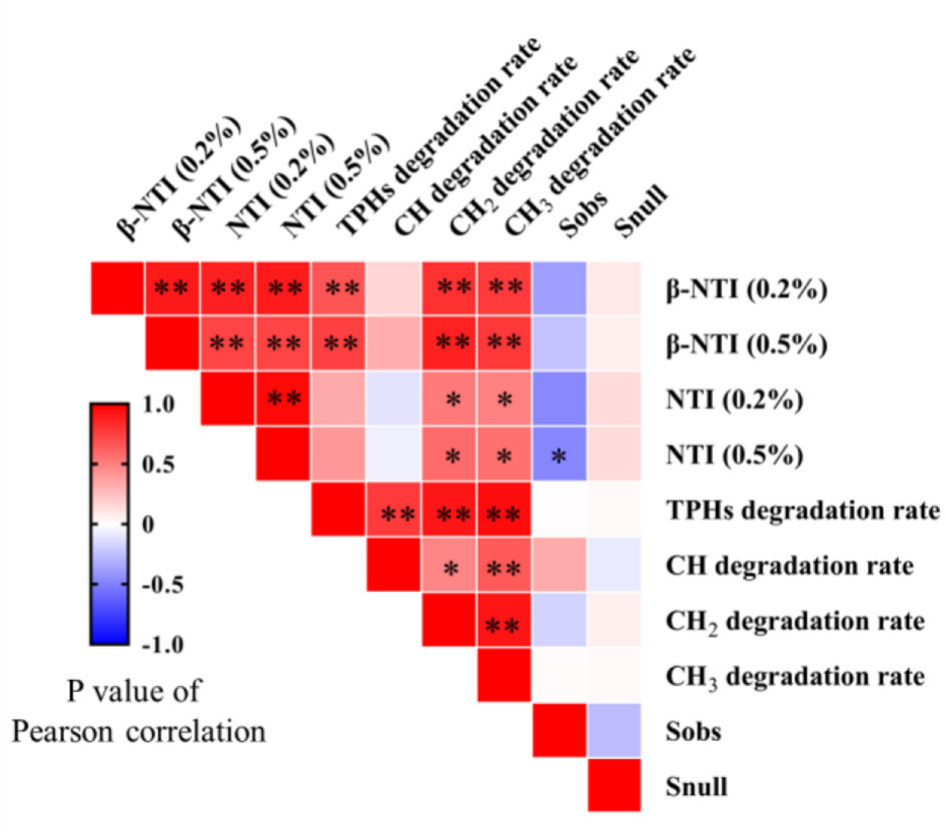
Pearson's correlation analysis between microbial community assembly process parameters and PHs' removal rate. The *P*-value and asterisk number were used to show significant differences where *P*>0.05, ns; *P* < 0.05, *; and *P* < 0.01, **.

The statistical correlation between microbial community assembly process parameters and PHs removal rate is shown in Figure 4. There was a significant positive correlation between NTI and CH_2_ and CH_3_ (*P* < 0.05). There was a significant positive correlation between β-NTI and TPHs, CH_2_, and CH_3_ removal rates (*P* < 0.01). The correlation between $\bar{S_{obs}}$ and $\bar{S_{null}}$ and the removal rate was poor, and the statistical correlation was not significant (*P* > 0.05).

## 4. Discussion

The community formation mechanism is critical for maintaining species distribution and diversity, and the theoretical study of community formation is one of the core topics in the field of environmental ecology (Ning et al., 2019). Although the fundamental laws of microbial diversity change are now well-understood, the factors influencing these laws remain unknown. As a result, environmental ecologists are focusing more on the formation of microbial communities and the process of community formation, which is a process of generating microbial diversity and community functions (Ning et al., 2020). In the field of microbial ecology, microbial community formation mechanisms are drawn from many macroeconomic theories and are divided into two major categories: deterministic processes and stochastic processes. Deterministic processes mainly consist of environmental factors, biological interactions, specialization, and priority effects, while stochastic processes include dispersal, birth, stochastic death, differentiation, specialization, and extrapolation (Zhou et al., 2013a,b). Regardless of which of these two processes dominates, community formations will determine the existence and abundance of species, thereby changing the diversity and composition of microorganisms, and have an impact on the function of the system (Feng et al., 2018).

The increase inaccumulation of PHs in the soil has caused changes of in the soil microecological environment, affecting the metabolism of microbial communities and changes in community composition and structure (Chen et al., 2022). When petroleum pollutants enter the soil, they not only have a toxic effect on the majority of soil microorganisms, but they also significantly reduce the number of active microorganisms in the soil, change the community structure, and cause uneven population distribution, resulting in a decrease in the function of microorganisms in the upper soil environment, which is manifested in a decrease in the activity of soil microorganisms (Zheng et al., 2020, 2023). Analyzing the structure–activity relationship between microbial communities and PHs removal efficiency is important for developing efficient microbial remediation techniques for PH-contaminated soil.

From the results of this study, it was discovered that the removal of PHs does not directly depend on the addition of exogenous microorganisms or surfactants (Figure 2); previous research also pointed out that the changes in the structure of indigenous microbial communities in the soil still have a significant impact on the removal of pollutants (Rong et al., 2021). The correlation among NTI, β-NTI, and PHs removal rate was significant and positive (Figure 4). Therefore, it is possible to conclude that the increase in microbial diversity caused by the deterministic assembly process can mediate the removal rate of PHs. Consequently, the study of microbial communities in the soil is critical for soil remediation research. The remediation of petroleum hydrocarbon-contaminated soil should not only focus on the removal of the pollutants but also on the impact of the remediation process on the diversity and function of the soil microbial community. At the same time, the study of the community formation process should further explore the mechanism of community diversity changes. There are many studies on remediation technology but very few on microbial communities after treatment. Only evaluating the removal rate of a remediation agent while ignoring its collateral effects on microbial communities in the soil cannot provide a comprehensive picture of the remediation process. Although some studies have shown that stochastic assembly can increase microbial diversity, which helps to generate more beneficial species and thus improve the functional diversity of microbial communities (Zhang et al., 2019), this is not consistent with the findings of this study. The deterministic assembly-induced microbial diversity is beneficial to improving the specific functions of microbial communities. This is likely due to increased stochastic, which leads to an increase in microbial species that do not have specific functions, competition with microbial species that do have specific functions, or changes in soil physical and chemical properties caused by their metabolites, which leads to a decline in the abundance of microbial species with specific functions. Therefore, the addition of remediation agents can effectively degrade PHs by regulating the deterministic assembly process of microbial communities in the soil, thereby stimulating microbial diversity.

## 5. Conclusion

This study calculated the assembly process of bacterial community structure under different treatments during microbial remediation of TPHs and analyzed the correlation with NTI, β-NTI, $\bar{S_{obs}}$ and $\bar{S_{null}}$, and the removal rate; it was discovered that the deterministic assembly process of bacterial communities may mediate the more efficient removal of TPHs. Based on the findings of this study, researchers should focus more on the targeted regulation of microbial ecological functions while treating contaminated soils using microbial-based remediation processes, rather than just the rough removal of pollutants through strong soil disturbance.

## Data availability statement

The original contributions presented in the study are included in the article/supplementary material, further inquiries can be directed to the corresponding author.

## Author contributions

XZ: methodology, writing—original draft, and funding acquisition. BO, LH, LF, JL, TD, and YD: writing—reviewing and editing. CS: writing—original draft and data curation. LR: data curation. BZ: validation. All authors contributed to the article and approved the submitted version.
